# The role of the Stop Transmission of Polio (STOP) program in developing countries: the experience of Kenya

**DOI:** 10.1186/s12889-020-09196-1

**Published:** 2020-07-14

**Authors:** Brook Tesfaye, Jeevan K. Makam, Kibet Sergon, Iheoma Onuekwusi, Charles Muitherero, Alieu Sowe

**Affiliations:** 1 World Health Organization, Kenya Country Office, United Nations Office in Nairobi (UNON), Gigiri Complex, Block U, Nairobi, Kenya; 2 The Centers for Disease Control and Prevention, Atlanta, Georgia United States of America

**Keywords:** Polio, Surveillance, Immunization, Stop Transmission of Polio (STOP), Human resource, Vaccine-Preventable Diseases (VPDs)

## Abstract

**Background:**

In 1988, the 41^st^ World Health Assembly (WHA) marked the launch of the Global Polio Eradication Initiative (GPEI) for the eradication of polio. A key component of the GPEI has been the development and deployment of a skilled workforce to implement eradication activities. In 1989, the Stop Transmission of Polio (STOP) was initiated to address skilled human resource gaps and strengthen poliovirus surveillance. This paper describes the role of the STOP 52 team in technical capacity building and health system strengthening in the implementation of polio eradication strategies in Kenya following the outbreak of Circulating Vaccine-derived Poliovirus type 2 (cVDPV2).

**Methods:**

Overview of the STOP program, deployment, and the modality of support are described. Descriptive analysis was conducted using data collected by the STOP 52 team during integrated supportive supervisory visits conducted from July 2018 to September 2019. Analyses were carried out using Epi-Info statistical software (Version 7.0) and maps were developed using Quantum Geographic Information System (Q-GIS) (version 3.12.0).

**Results:**

The STOP 52 team supportively supervised 870 health facilities on Expanded Program on Immunization (EPI), and Acute Flaccid Paralysis (AFP) and other Vaccine-Preventable Diseases (VPDs) surveillance in 16 (34.1%) of the 47 counties during the study period. AFP surveillance was conducted in all health facilities supervised leading to the detection and investigation of 11 unreported AFP cases. The STOP 52 team, as part of the outbreak response, provided technical support to five successive rounds of polio Supplementary Immunization Activities (SIAs) conducted during the study period. Moreover, in addressing programmatic data needs, the STOP 52 Data Manager played a valuable role in enhancing the quality and use of data for evidence-based planning and decision-making. The STOP 52 team contributed to the development of operational plans, guidelines and training manuals, and participated in the delivery of various Training of Trainers (TOT) and On-the-Job Training (OJT) on EPI, AFP and other VPDs surveillance including data management.

**Conclusion:**

The STOP 52 team has contributed to polio eradication efforts in Kenya by enhancing AFP and other VPDs surveillance, supporting polio SIAs, strengthening EPI, use of quality EPI, AFP and other VPDs data, and capacity building of Frontline Health Workers (FLWs). The use of Open Data Kit (ODK) technology during supportive supervision, and AFP and other VPDs surveillance was found to be advantageous. A national STOP program should be modeled to produce a homegrown workforce to ensure the availability of more sustainable technical support for polio eradication efforts in Kenya and possibly other polio-affected countries.

## Background

Poliomyelitis, commonly known as Polio, is a contagious disease caused by the poliovirus [1] that attacks the Central Nervous System (CNS) [2]. As a highly infectious disease, the poliovirus is transmitted through contact with infected fecal matter entering the oral route [2]. The Wild Poliovirus (WPV) occurs in three serotypes, type 1, type 2, and type 3 [3]. However, WPV2 and WPV3 were eradicated in 1999 and 2019 respectively [4]. Pakistan, Afghanistan, and Nigeria are the only countries considered not free from WPV1 [4]. However, no WPV1 case has been detected in Nigeria since 2012 [4].

In Kenya, the last case of WPV1 was detected in 2013. This case was an importation from neighboring Somalia and had a date of onset of paralysis of 14^th^ July 2013. The most recent outbreak was a Vaccine-derived Poliovirus type 2 (VDPV2) isolate from an environmental sample, Kamukunji site 2, in the Eastleigh area of Nairobi. The sample was collected on the 21^st^ of March 2018. Sequencing results received on 11^th^ April 2018 confirmed it was a circulating Vaccine-derived Poliovirus type 2 (cVDPV2) with 47 nucleotide differences from the parent Sabin 2 strain. This strain was genetically linked to environmental sample isolates detected in Benadir, Somalia, in October and November 2017.

In 1988, the World Health Assembly (WHA) established the Global Polio Eradication Initiative (GPEI), a partnership between the World Health Organization (WHO), Rotary International (RI), the Centers for Disease Control and Prevention (CDC), the United Nations Children’s Fund (UNICEF), and national governments aiming to achieve a polio-free world [5]. Later, the Bill and Melinda Gates Foundation (BMGF) and the Global Alliance for Vaccines and Immunizations (GAVI) also joined the GPEI. The GPEI underlined four proven strategies to eradicate polio worldwide [6]. These are: 1) maintaining high population immunity using Oral Polio Vaccine (OPV) and Inactivated Polio Vaccine (IPV) through the Expanded Programme on Immunization (EPI), 2) detect and interrupt the circulation of all suspected cases of Poliomyelitis through sensitive Acute Flaccid Paralysis (AFP) surveillance, 3) Supplementary Immunization Activities (SIAs), and 4) mop-up campaigns [6, 7].

In the perspective of fulfilling the skilled human resource gap required for the effective implementation of GPEI strategies to eradicate polio, the Stop Transmission of Polio (STOP) program was designed as a key component of the GPEI [8] and its implementation started in 1998 in polio-endemic countries - Afghanistan, Pakistan, and Nigeria [9]. The program was later expanded to include other Vaccine-Preventable Diseases (VPDs) surveillance, routine immunization, communication, and data management [8, 9]. Consequently, in 1999, the first cohort of the STOP team (STOP 1), comprising of 25 experts, was assigned on a three-month field mission in six countries [10]. Later, the CDC started a recruitment process that allowed volunteer public health experts from around the world to gain useful experience and contribute to polio eradication efforts [8–11].

The objective of this study is to assess core activities and major achievements of the STOP 52 team in supporting the implementation of GPEI activities in Kenya from July 2018 to September 2019.

## Methods

### Study area, design and period

Kenya is located in East Africa and the country has forty-seven semi-autonomous counties, which are further divided in to sub counties. Retrospective secondary data analysis was carried out t using data collected by the STOP 52 team during supervisory visits conducted from July 2018 to September 2019.

### The STOP 52 team composition and Terms of Reference (TOR)

The STOP 52 team was composed of four Field Epidemiologists and one Data Manager, who successfully completed a three-week pre-deployment specialized training on EPI, and AFP and other VPDs surveillance provided by GPEI partners.

The Terms of Reference (TOR) were developed considering GPEI polio eradication strategies and country specific needs at different levels of the Kenyan health system. The TOR included 1) enhancing AFP and VPDs surveillance system, 2) strengthening EPI, 3) support implementation of polio SIAs, and 4) improving EPI, AFP and other VPDs surveillance data management, and 5) health system strengthening and local capacity building.

### STOP 52 team deployment strategy

Counties of deployment for the STOP team were selected based on AFP surveillance and routine immunization performance. Counties that had suboptimal performance were selected and each Field Epidemiologist was deployed to one county for a period of six weeks. However, during polio SIAs, the STOP 52 team was re-assigned to counties targeted for SIAs. The STOP 52 Data Manager was permanently stationed at the central level, WHO Kenya Country Office and the national Ministry of Health.

### STOP 52 team liaison and partnerships

Since its inception, the STOP program has mobilized human resources and deployed them to support national ministries of health, WHO, and UNICEF activities [10]. Thus, liaison and partnerships have been critical to the success of the program. The STOP 52 team members were officially under the supervision of the WHO Kenya Country Office. WHO officers guided STOP 52 team members in developing work plans, liaised with Ministry of Health counterparts to make sure the work plans address national priorities, and supervised their core activities. Each STOP 52 team member was required to submit a bi-weekly work plan to their field supervisor for review and approval. After every two weeks, all STOP 52 team members were also expected to submit written bi-weekly reports of the activities conducted.

### Data source

The data for this study were retrieved from the WHO Integrated Supportive Supervision (ISS) database. The ISS checklist is, an android based real-time data collection checklist built using the Open Data Kit (ODK) tool, used by the STOP 52 team and other officers such as WHO surveillance and immunization officers, Ministry of Health, and other developmental partners during supportive supervision visits. After completion of each supervision visit, the data collected is submitted in real-time to the WHO central server.

### Data analysis and presentations

The database was downloaded in Microsoft Comma-separated values (MS CSV) file format. Prior to analysis, data quality was assessed and only data submitted by the STOP 52 team were extracted. Descriptive data analysis was conducted using Epi-Info statistical software (version 7; CDC, Atlanta, United States) and maps were developed using Quantum Geographical Information System (Q-GIS) (version 3.12.0). Findings of the study were presented using tables and maps.

## Results

### Contribution to enhancing AFP surveillance

**Fig. 1:**
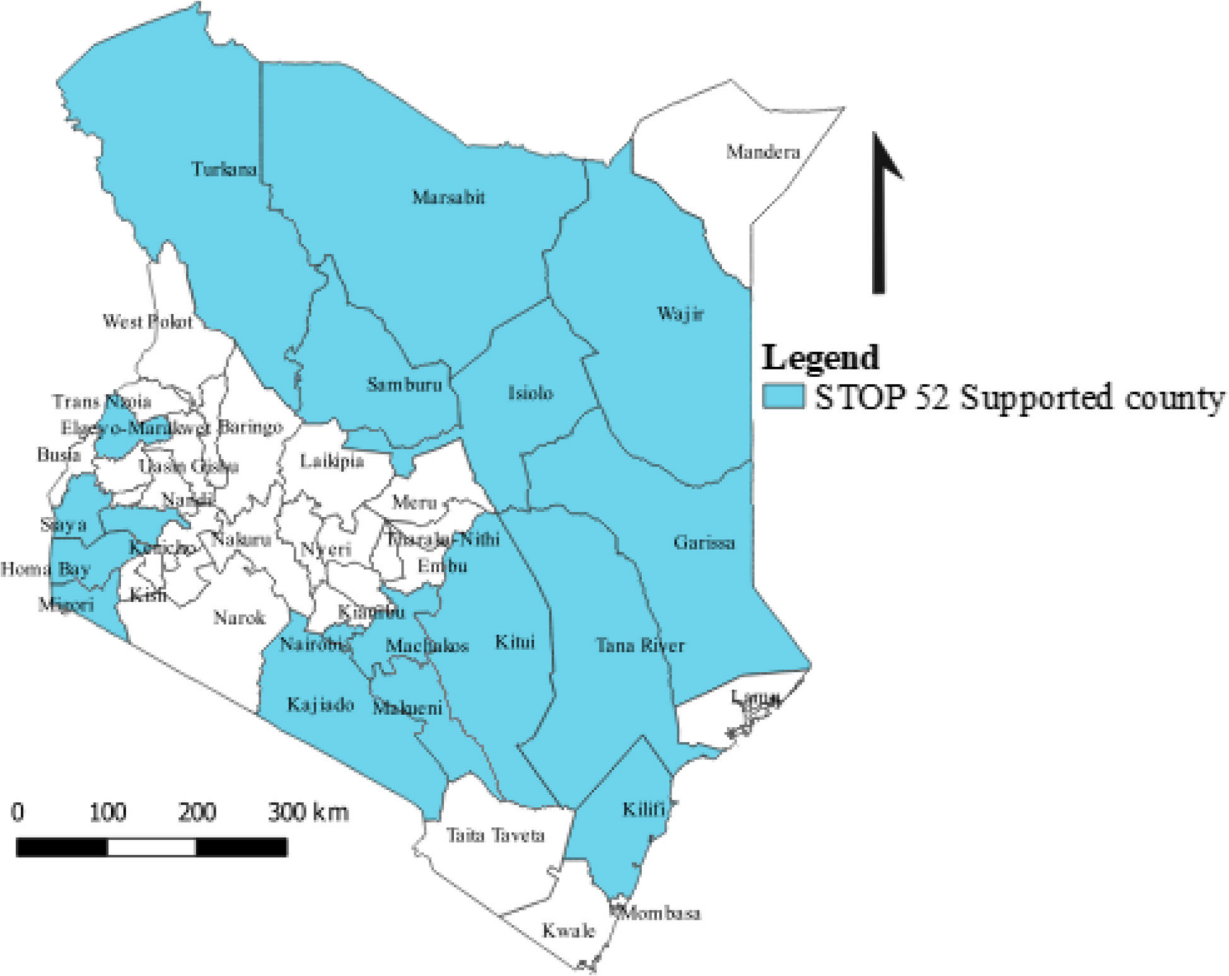
Counties supported by the STOP 52 team, Kenya, July 2018 – September 2019. Source: The maps were developed by authors using Quantum Geographic Information System (Q-GIS) (version 3.12.0)

**Fig. 2:**
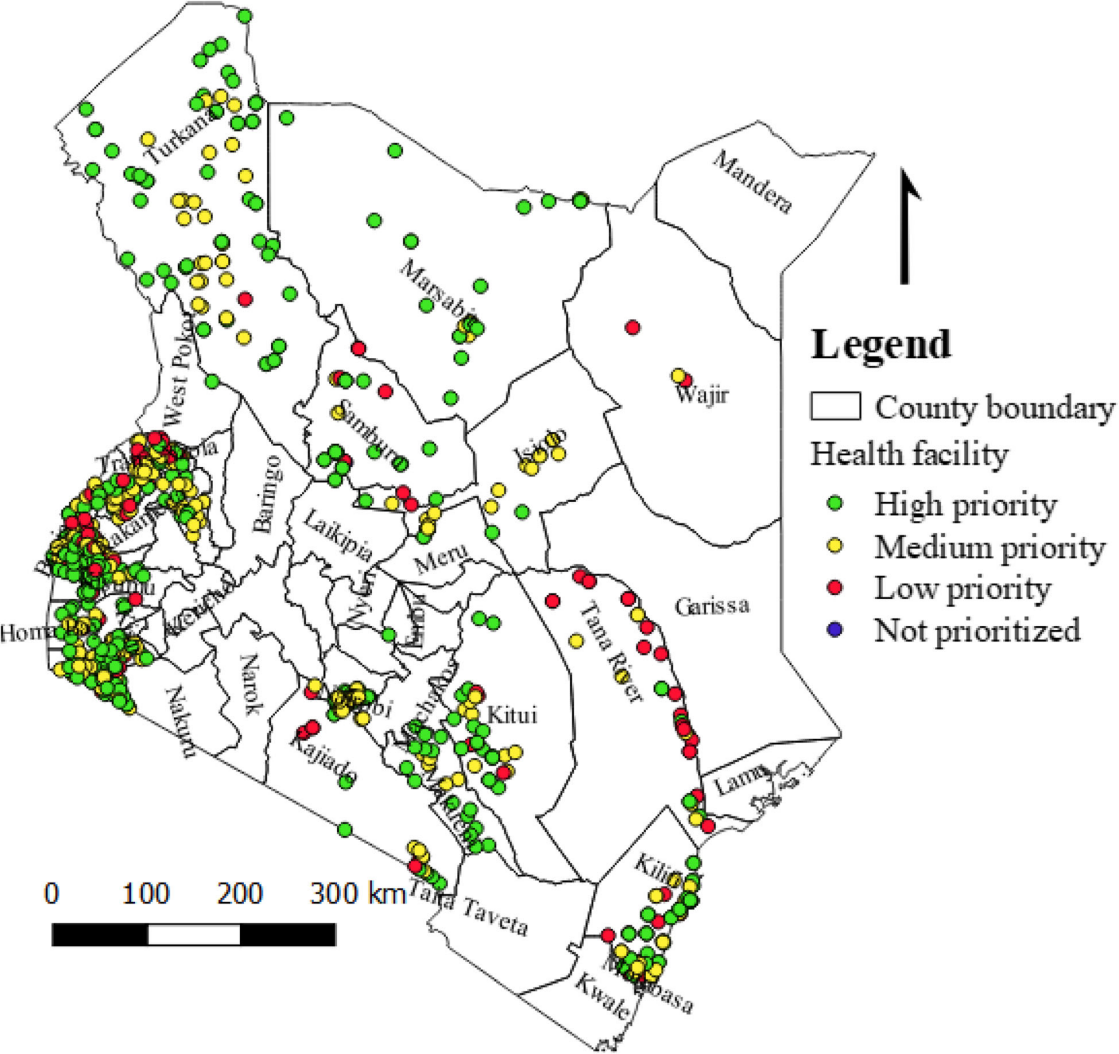
Geo-coordinates and polio surveillance prioritization of supervised health facilities by the STOP 52 team, Kenya, July 2018 – September, 2019. Source: The maps were developed by authors using Quantum Geographic Information System (Q-GIS) (version 3.12.0)

STOP 52 Field Epidemiologists, between July 2018 and September 2019, conducted active surveillance for AFP and other VPDs in 870 health facilities in 16 (30.7%) of the 47 counties (Fig. 1). Of these facilities, 273 (31.4%) were health centers, 215 (24.7%) were dispensaries, 164 (18.8%) were hospitals, 117 (13.4%) were clinics and the remaining 11.7% were other health facility types (military, bonesetters, traditional healers). By ownership, majority (85.8%) of the health facilities were government health facilities. Nearly half (49.4%) of active surveillance visits for AFP were conducted in health facilities classified as high priority for detecting an AFP case. Two hundred and sixty-one (30.0%) and 131 (15.1%) health facilities were classified as medium and low priority respectively and the remaining 5.5% health facilities were unclassified. Eleven missed cases of AFP were detected and reported by the STOP 52 team. Geographical locations for all health facilities where active surveillance were conducted were captured (Fig. 2).

Seven hundred and eighty-three (90.0%) active surveillance visits were conducted jointly by the STOP 52 team and Ministry of Health disease surveillance focal persons. Three hundred and ninety one (44.9%) health facility surveillance focal persons were trained on VPDs surveillance focused on AFP, measles, Neonatal Tetanus (NNT) and yellow fever, in less than a year from supervision dates. Six hundred and twenty-five (71.8%) health facility surveillance focal persons knew the case definition for AFP and 523 (60.1%) knew specimen collection procedures for a suspected AFP case. Of the supervised health facilities, 561 (64.5%) had posters with standard case definitions of VPDs including AFP. About a quarter (224) of the health facilities supervised had guidelines for AFP surveillance. Seven hundred and five (81.0%) health facility surveillance focal persons had a list of community informants. However, 147 (16.9%) health facility surveillance focal persons never sensitized their community informants on AFP surveillance.

Other activities the STOP Field Epidemiologists conducted, supported or facilitated in the field included case investigations, AFP case validation, 60-day follow-up, geocoding AFP cases, contact sampling for AFP cases requiring so, and surveillance related On-the-Job Trainings (OJT) and sensitization of health workers, community health workers, and traditional healers.

### Contribution to strengthening routine immunization

Of the total 870 health facilities supervised by the STOP 52 team, 792 (91.4%) provide immunization services. As depicted in Table 1, of the fixed and outreach routine immunization sessions planned in these health facilities, 93.2% fixed and 67.5% outreach sessions were implemented. Routine immunization sessions were interrupted in 219 (27.6%) health facilities. Expired vaccines, Vaccine Vial Monitor (VVM) beyond stage 2, and reconstituted freeze-dried vaccines were found in 12 (1.5%), 19 (2.4%) and 8 (1.1%) of the health facilities supervised respectively. OPV stock-out was reported in 69 (8.7%) health facilities, and Adverse Events Following Immunization (AEFI) were reported in 14 health facilities (Table 1). The STOP 52 team conducted OJT on routine immunization for health workers to address knowledge gaps and took necessary immediate steps to correct adverse findings during supportive supervisory visits where applicable.

**Table 1:** Findings on routine immunization supportive supervision using Integrated Supportive Supervision (ISS) tool, Kenya, July 2018 – September 2019

Characteristics	Yes		No	
	N	%	N	%
Availability of updated schedule for routine immunization sessions	686	86.6%	106	13.4%
Fixed routine immunization sessions implemented	738	93.2%	54	6.8%
Outreach routine immunization sessions implemented	535	67.5%	257	32.5%
Interrupted routine immunization sessions	219	27.6%	573	72.4%
Availability of updated routine immunization monitoring chart	422	53.3%	370	46.7%
Availability of immunization related Information Education and Communication (IEC) materials	68	8.6%	724	91.4%
Availability of functional fridge	765	96.6%	27	3.4%
OPV in good condition (VVM stage 1 and 2)	703	88.7%	89	11.3%
Knowledge on VVM reading	728	91.9%	64	8.1%
Knowledge on vaccine shake test	264	33.3%	528	66.7%
OPV stock out	546	69.0%	246	31.0%
AEFI reporting	111	14.0%	681	86.0%
Availability of vaccine ledger book	732	92.4%	60	7.6%
Availability of funds for strengthening routine immunization for the last 1 year	339	42.8%	453	57.2%

### Support to the implementation of polio SIAs

Kenya implemented five successive rounds of polio SIAs between July 2018 and September 2019. The SIAs were in response to the cVDPV2 outbreak in Kamukunji Sub County, Nairobi County. The STOP 52 team played an important role in the implementation of all five SIA rounds. They supported activities such as microplanning and training of team supervisors and vaccination teams on developing team movement plans, polio vaccine administration procedures, cold-chain management including VVM reading, and data recording and reporting using tally sheet and android-based data collection tools. The STOP 52 team conducted pre-campaign and intra-campaign monitoring, which helped to identify gaps and implement immediate corrective actions while the campaign process was underway. In the perspective of assessing the quality of SIAs implemented, the STOP 52 team supported Independent Monitoring (IM) and Lot Quality Assurance Sampling (LQAS) activities by training and supervising IM monitors and LQAS surveyors in deployment counties. The team also supported and participated in national, county, and sub-county SIAs review meetings.

### Contribution towards improving routine immunization, AFP and other VPDs surveillance data management

The STOP 52 Data Manager, between July 2018 and September 2019, contributed to the production and dissemination of 60 polio surveillance weekly Situational Reports (SitReps). The reports presented the total number of AFP cases with their epidemiological distribution, performance on key polio surveillance indicators, including laboratory, and status updates on key polio surveillance activities. Similarly, he supported on measles and routine immunization data cleaning, analysis, presentation, and dissemination. The STOP 52 team Data Manager contributed to the development and costing of AFP surveillance annual operational plans, including target setting, at national and sub-national levels. He contributed in the preparation of various technical reports and presentations. One annual measles and 5 quarterly polio risk assessments were conducted and results were shared with program managers. Using ODK technology, the STOP 52 team Data Manager supported the development of various android-based real-time data collection and reporting tools to supplement immunization and VPDs surveillance program improvement activities, and provided subsequent training on how to use them. He analysed real-time intra-campaign data and provided timely feedback to the national and sub-national levels during all 5 rounds of polio SIAs. Furthermore, he collaborated on administrative, IM, and LQAS data cleaning, analysis, and presented findings during national review meetings. The STOP 52 data manager collaborated in delivering TOT to county disease surveillance coordinators on AFP and other VPDs surveillance including data management. He also shared his knowledge and skills on application of various data analysis software tools with WHO and Ministry of Health data managers.

## Discussion

Sub-optimal programmatic performance driven by a lack of skilled human resource required for polio eradication have been identified as major challenges in polio-affected countries [10, 12]. The STOP program, a network of skilled public health professionals who could provide sustainable support to the polio eradication efforts [10], including knowledge and skill transfer, is one pillar of the GPEI [13]. This study demonstrated the role of the STOP 52 team in strengthening polio eradication activities in Kenya. It highlighted major activities the team contributed to and success achieved; emphasizing local health workforce development and health system strengthening for sustainability.

During the study period, the STOP 52 team enhanced active surveillance by actively searching for and reporting cases that met standard case definitions [14], which is the primary means of detecting the poliovirus [15]. The team was able to detect missed AFP cases at health facilities and the community. This has improved the sensitivity of AFP surveillance by increasing the non-polio AFP detection rate. In addition, all active surveillance visits were conducted in collaboration with county and sub-county disease surveillance coordinators which paved a way for knowledge and skill transfer and fostered local capacity building that will enhance the basics of AFP surveillance.

One of the core strategies employed by the GPEI to eradicate the polio disease is strengthening routine immunization to achieve high vaccination coverage with quality polio vaccines [3]. Cognizant of this, the STOP 52 team played a key role in detecting and improving cold-chain gaps and vaccine stock-out. The team conducted OJT for local health staff whenever they found immunization and cold-chain related gaps to prevent recurrence. Since the STOP 52 team members were almost always accompanied by at least one officer from the county or sub-county level, gaps that should be addressed by the county or sub-county level where noted and addressed in the shortest possible time.

The STOP 52 team participated in the development of various operational plans, guidelines, tools, and training materials. The team coordinated and provided various national and sub-national level trainings including OJT on VPDs surveillance, routine immunization, and programmatic data management. Supportive supervisions has been found to be effective methods in improving not only AFP surveillance system, but also other public health programs [16]. It helped the local health workforce to consistently improve their performance, share knowledge and skills, and solve other systematic problems that contribute to suboptimal programmatic performance [17].

The STOP 52 team supported the local staff in mapping under-served populations and inaccessible areas to reach children who may be underserved by the surveillance system and routine immunization program. This activity in parallel with other strategies such as Community Based Surveillance (CBS) where Community Health Volunteers (CHVs) report suspected cases of AFP enhance the capacity of the AFP surveillance system in detecting more AFP cases [18]. In this regard, the STOP 52 Field Epidemiologists played a key role in sensitizing CHVs on AFP and VPDs surveillance.

The STOP 52 team provided technical assistance to county and sub-county diseases surveillance coordinators on investigating and validating AFP cases, conducting 60 days follow-up examination, and contact sampling. They also supported the investigation of AFP cases with zero OPV doses, missing age, and unknown immunization status. Such support helped prevent costs related to contact sampling and 60 days follow-up that would have been incurred if prior appropriate investigations with complete information were not carried out.

The STOP 52 team contributed to strengthening surveillance performance monitoring by encouraging county and sub-county diseases surveillance coordinators to track key performance indicators on a regular basis through effective data use [19]. Part of the monitoring they encouraged also includes the tracking of silent sub-counties. A silent sub-county is one that did not report an AFP case in a period varying from six to twelve months or more, depending on their target population. To this end, the STOP 52 team guided county diseases surveillance coordinators in mapping and developing appropriate strategies to enhance AFP and other VPDs surveillance in silent sub-counties. All strategies followed GPEI procedures including rapid assessment in search of potential surveillance gaps, triggering active case search and strengthening CBS (including sensitization of CHVs and traditional healers) to strengthen the surveillance network.

The use of ODK technology during active surveillance was found to be advantageous by the STOP 52 team. The STOP 52 team used android-based ODK technology to collect, aggregate, and report real-time data, including geospatial data, during active surveillance. This played a vital role in improving surveillance data quality and information use for informed decision making at the national and sub-national levels [13, 19, 20]. The technology was also found more reliable and seemingly more cost-effective for AFP surveillance by averting costs related to paper-based data collection methods. A study in Nigeria [21] and Thailand [22] also upheld similar findings.

## Conclusions

The STOP 52 team consultants have contributed to polio eradication efforts in Kenya by enhancing AFP and other VPDs surveillance, supporting polio SIAs, strengthening EPI, and improving use of quality immunization and surveillncae data. The team has played a valuable role in sharing skills, knowledge, and building the capacity of the local workforce through trainings and supportive supervisions at different levels of the health system. A national STOP program should be modeled to produce homegrown workforce for more sustainable support for polio eradication efforts in Kenya and possibly other polio-affected countries. Supportive supervision and active surveillance should be strengthened especially in hard-to-reach health facilities and high-risk areas where the likelihood of undetected poliovirus transmission may be higher.

## Data Availability

The data used for this study can be accessed from the World Health Organization, Kenya Country Office with a justifiable request.

## Abbreviations

AFP: Acute Flaccid Paralysis
cVDPV2: circulating Vaccine-derived Poliovirus type 2
ISS: Integrated Supportive Supervision
ODK: Open Data Kit
OJT: On-the-Job Training
SIAs: Supplementary Immunization Activities
VDPV2: Vaccine-derived Poliovirus type 2
VPDs: Vaccine-Preventable Diseases
WPV: Wild Poliovirus
